# Effects of herbal nutraceuticals and/or zinc against *Haemonchus contortus* in lambs experimentally infected

**DOI:** 10.1186/s12917-018-1405-4

**Published:** 2018-03-09

**Authors:** Zora Váradyová, Dominika Mravčáková, Michal Babják, Magdalena Bryszak, Ľubomíra Grešáková, Klaudia Čobanová, Svetlana Kišidayová, Iveta Plachá, Alžbeta Königová, Adam Cieslak, Sylwester Slusarczyk, Lukasz Pecio, Mariusz Kowalczyk, Marián Várady

**Affiliations:** 1 Institute of Animal Physiology, Centre of Biosciences, Slovak Academy of Sciences, Šoltésovej 4-6, 040 01 Košice, Slovak Republic; 2 Institute of Parasitology, Slovak Academy of Sciences, Hlinkova 3, 040 01 Košice, Slovak Republic; 3 Department of Animal Nutrition and Feed Management, Poznan University of Life Sciences, Wolynska 33, 60-637 Poznan, Poland; 4 Department of Biochemistry, Institute of Soil Science and Plant Cultivation, State Research Institute, Czartoryskich 8, 24-100 Pulawy, Poland; 5 Department of Pharmaceutical Biology with Botanical Garden of Medicinal Plants, Medical University of Wroclaw, Wroclaw, Poland

**Keywords:** Anthelmintic activity, Egg counts, Gastrointestinal nematode, *Haemonchus contortus*, Herbal bioactive compounds, Mineral status, Organic zinc, Sheep

## Abstract

**Background:**

The gastrointestinal parasitic nematode *Haemonchus contortus* is a pathogenic organism resistant to several anthelmintics. This study assessed the efficacy of a medicinal herbal mixture (Herbmix) and organic zinc, as an essential trace element for the proper functioning of both unspecific and specific immune defensive mechanisms, against experimental infections with *H. contortus* in lambs. All lambs were infected orally with approximately 5000 third-stage larvae of a strain of *H. contortus* susceptible to anthelmintics (MHco1). Twenty-four female lambs 3–4 months of age were divided into four groups: unsupplemented animals (control), animals supplemented with Herbmix (Hmix), animals supplemented with organic zinc (Zn) and animals supplemented with Herbmix and organic zinc (Hmix+Zn). Eggs per gram (EPG) of faeces were quantified 20, 28, 35, 42, 49, 56, 62 and 70 d post-infection and mean abomasal worm counts were assessed 70 d post-infection. Samples of blood were collected from each animal 7, 35, 49 and 70 d post-infection.

**Results:**

Quantitative analyses of the bioactive compounds in Herbmix identified three main groups: flavonoids (9964.7 μg/g), diterpenes (4886.1 μg/g) and phenolic acids (3549.2 μg/g). Egg counts in the lambs treated with Hmix, Zn and Hmix+Zn decreased after 49 d. The EPGs in the Zn and Hmix+Zn groups were significantly lower on day 56 (*P* < 0.05 and *P* < 0.01, respectively), and the EPGs and mean worm counts were significantly lower on day 70 in all supplemented groups (*P* < 0.05 and *P* < 0.01). Hemograms of complete red blood cells of each animal identified clinical signs of haemonchosis after day 35. Serum calprotectin concentrations and IgA levels were significantly affected by treatment. The treatment influenced serum malondialdehyde concentrations (*P* < 0.05) and sulfhydryl groups (*P <* 0.01) of antioxidant status. The mineral status was unaltered in all lambs.

**Conclusion:**

A direct anthelmintic impact on the viability of nematodes was not fully demonstrated, but the treatments with herbal nutraceuticals and zinc likely indirectly contributed to the increase in the resistance of the lambs to nematode infection.

## Background

Plant secondary metabolites (PSMs) of medicinal herbs, which have been used for centuries in traditional medicine and veterinary practice to treat various digestive or parasitic disorders, have important biological activities [1–3]. PSMs are bioactive compounds with applications in nutraceuticals and functional food. Compounds with nutraceutical activity [4, 5] however, are mostly produced chemically, but new demands for a sustainable life are gradually favoring natural compounds, mainly those derived from herbs [6]. A recent study [7] has summarized information from 2001 to 2016 on polyphenols demonstrating promising anthelmintic activities of polyphenols that can be used as an additional or alternative treatment to current standard anthelmintics and as a base for the development of new substances that are urgently needed in human and veterinary medicine.

Chemoprophylaxis against the gastrointestinal parasitic nematode *Haemonchus contortus* by the repeated application of anthelmintics increases the risk of residues in food products and the development of anthelmintic resistance. Studies of foods containing healthy additives and having medicinal benefits in parasitized ruminants indicate that herbs with bioactive compounds represent a promising option for reducing nematode infections in small ruminants and for applications under farm conditions [8–10]. Phytotherapeutic treatments in traditional medicine [11] combined with the pharmacology of PSMs [12] and self-medication of ruminants against gastrointestinal parasites [13] can be used as an alternative strategy to control gastrointestinal helminths of small ruminants. The ability of the host to resist infection by gastrointestinal nematodes, though, is dependent on the development of a protective acquired immune response [14]. Nematode infections may also induce the production of reactive oxygen species, which may damage the parasites but generate oxidative stress in the hosts [15]. The trace element zinc is essential for the proper functioning of both unspecific and specific immune defensive mechanisms and reduces the impact of many diseases by preventing disassembly of the immune system [16, 17]. Zinc is essential for the improvement of the immune functions because the efficiency of immunological responses, mainly intestinal immunity, against gastrointestinal nematodes depends on the zinc status and Zn nutrition [17]. The type of diet and availability of minerals are directly associated with the susceptibility of animals to parasites. Targeted nutrition and in vitro protocols for the mass production of PSMs have received much attention, but little emphasis has been placed on nutraceutical activity and analysis [6]. Supplementing diets with both medicinal herbs and zinc has therefore been hypothesized to affect the life cycle of *H. contortus*, have a direct anthelmintic impact on the viability of nematodes and may provide a better defense against oxidative stress of lambs, with an indirect impact on an increase in the resistance of hosts to parasitic infections of *H. contortus*. In this study the mix of traditional medicinal herbs (Herbmix) consist of herbs typical for Central Europe. These herbs were chosen based on information about their phytotherapeutic properties from traditional ethnomedicine practice.

Our goals were to (1) identify the main bioactive compounds of a medicinal herbal mixture (Herbmix) and (2) determine the effect of dietary supplementation with Herbmix and organic zinc (Zn-glycinate) on mean live-weight gain, parasitological status, hematological parameters, inflammatory response, antioxidative status and mineral status of lambs experimentally infected with *H. contortus*.

## Methods

### Animals, diets and experimental design

Twenty-four female lambs (Improved Valachian) 3–4 months of age with initial body weights of 15.12 ± 1.58 kg were housed in common stalls for 7 d for acclimatization to feeding, with free access to water. Each animal was fed a concentrate (500 g dry matter (DM)/d), Herbmix (a non-commercial product, 100 g DM/d) and meadow hay (ad libitum). The concentrate was composed of 37% wheat bran, 20% soybean meal, 23% rolled oats and 20% maize meal. After this adaptation period, all parasite free lambs were separated into four distinct pens, infected orally with approximately 5000 third-stage (L3) larvae of a strain of *H. contortus* susceptible to anthelmintics MHco1 (MOSI) which is susceptible to all the main classes of anthelmintics. It has been maintained since the late 1950s, and is thought to have been isolated in East Africa. Animals were randomly divided based on their live-weight into four groups of six animals each (*n* = 6/group, one stall per group): unsupplemented animals (control, C), animals supplemented with Herbmix (Hmix), animals supplemented with a zinc chelate of glycine hydrate - Glycinoplex-Zn 26% (Zn) and animals supplemented with both Herbmix and zinc (Hmix+Zn). The number of animals used in experiment was assigned according to VICH GL13 guidelines. The experimental period lasted 70 days (during summer) and the animals were housed in sheep farm. Herbmix is a mixture of dry herbs obtained from commercial sources (AGROKARPATY, Plavnica, Slovak Republic and BYLINY Mikeš s.r.o., Číčenice, Czech Republic): roots of marshmallow (*Althaea officinalis* L.), butterbur (*Petasites hybridus* L.) and elecampane (*Inula helenium* L.); leaves of ribwort plantain (*Plantago lanceolata* L.) and rosemary (*Rosmarinus officinalis* L.); seeds of fennel (*Foeniculum vulgare* Mill*.*) and stems of goldenrod (*Solidago virgaurea* L.), fumitory (*Fumaria officinalis* L.) and hyssop (*Hyssopus officinalis* L.). The Herbmix contained 11.8% each of *A. officinalis*, *P. hybridus*, *I. helenium*, *P. lanceolata*, *R. officinalis*, *S. virgaurea*, *F. officinalis* and *H. officinalis* and 5.6% *F. vulgare*. The Herbmix was stable throughout the experiment, mixed daily with the commercial concentrate from day D7 to D70. Aliquots of the zinc supplement were directly mixed with the concentrate for each feeding to provide an additional 60 mg zinc/kg concentrate. The allowed upper limits of zinc in complete feed is 120 mg/kg for food-producing animals [18]. Herbal and zinc supplementation began on D7. The chemical compositions of Herbmix, meadow hay, commercial concentrate and concentrate + Glycinoplex-Zn are given in Table 1. The lambs’ wool was clipped on D3. The lambs were weighed on D7, D35, D49 and D70. Faeces were collected from the rectum, and the number of eggs per gram (EPG) of faeces was quantified on D0, D20, D28, D35, D42, D49, D56, D62 and D70. Samples of blood were collected from each animal on D7, D35, D49 and D70.

**Table 1:** Chemical composition of the diet substrates and main Herbmix phytochemicals

Substrate	DM	NDF	ADF	CP	N	Ash	Zinc	Phenolic acids	Diterpenes	Flavonoids
	(g/kg)	(g/kg DM)	(g/kg DM)	(g/kg DM)	(g/kg DM)	(g/kg DM)	(mg/kg)	(mg/g DM)	(mg/g DM)	(mg/g DM)
Herbmix	905	532	452	207	33	84	26.2	3.55	4.89	9.96
Meadow hay	900	651	556	163	27	91	66.3	n.d.	n.d.	n.d.
Concentrate	878	136	83	309	49	29	41.1	n.d.	n.d.	n.d.
Concentrate + zinc	876	254	93	352	56	30	88.8	n.d.	n.d.	n.d.

*DM* dry matter, *NDF* neutral-detergent fiber, *ADF* acid-detergent fiber, *CP* crude protein, *N* nitrogen, *Ash* mineral matter present in feed, *n.d.* not determined

### Chemical measurement and analysis

The dietary substrates (Herbmix, meadow hay, concentrate and concentrate + Zn-glycinate) were analyzed in triplicate for DM (No. 967.03), ash (method no. 942.05) nitrogen (method no. 968.06) and crude protein (method no. 990.03) using standard methods as described by AOAC [19]. The acidic-detergent fiber and neutral-detergent fiber contents were analyzed as described by Van Soest et al. [20] using an ANKOM 2000 fiber analyzer (ANKOM Technology, Macedon, USA) with heat-stable α-amylase.

### Analysis of phenolic acids, flavonoids and diterpenes

The plant samples were ground to a fine powder, and 100 mg were extracted three times with a Dionex ASE (Accelerated Solvent Extractor, ThermoFisher, Sunnyvale, CA, USA) in 80% MeOH for 15 min (three static cycles, 5 min each), at 1500 *psi* solvent pressure, 100 °C cell temp., flush 150%. The extracts were evaporated to dryness, dissolved in 1 mL of Milli-Q water (acidified with 0.2% formic acid) and purified by Solid Phase Extraction (SPE) using C18 Sep-Pak cartridges (1 cm^3^, 360 mg, Waters Corp., Milford, MA). The cartridges were washed with 0.5% methanol to remove carbohydrates, and then washed with 80% methanol to elute phenolics. The phenolic fraction was evaporated and dissolved in 1 mL of 80% methanol (acidified with 0.2% formic acid). The sample (intense yellow color without chlorophyll) was than centrifuged (18,766×*g*, 5 min) before spectrometric analysis. All analyses were performed in triplicate for three independent samples and stored in a freezer at − 20 °C before analysis.

### Ultra-high-resolution mass spectrometry (UHRMS)

The Herbmix bioactive compounds were analyzed by UHRMS on a Dionex UltiMate 3000RS (Thermo Scientific, Darmstadt, Germany) system with a charged aerosol detector interfaced with a high-resolution quadrupole time-of-flight mass spectrometer (HR/Q-TOF/MS, Impact II, Bruker Daltonik GmbH, Bremen, Germany). The Herbmix metabolome was chromatographically separated on an Acquity UPLC BEH C18 column (100 × 2.1 mm, 1.7 μm, Waters, Manchester, UK) maintained at 50 °C. The mobile phase consisted of: A (0.1% formic acid in Milli-Q water, *v*/v) and B (0.1% formic acid in acetonitrile, v/v) at a flow rate of 0.4 mL/min. The gradient elution was: 7% B from 0 to 0.5 min with a short 0.3 min calibration segment, and the concentration of B was then increased to 70% from 0.5 to 17 min. The column was eluted with this concentration of solvent B for 0.5 min and was then re-equilibrated for 0.2 min at a flow rate of 0.5 mL/min at 50 °C. The samples were kept at 15 °C in the autosampler. The injection volume was 5.0 μL. The mass spectrometer was operated in the positive atmospheric-pressure chemical ionization (APCI) mode after confirmation of low sensitivity and poor resolution in the negative mode. An APCI tuning mix (Agilent, Santa Clara, CA, USA) (Pos) in quadratic mode and locked mass was used for calibration with the following parameters: capillary voltage was set at 2.8 kV, nebulizer 0.7 bar, dry gas 6.0 L/min and dry temperature 200 °C. The mass scan range was set at 50–1870 m/z. MS/MS spectra were acquired in a data-dependent manner, whereby ions (maximum 2) from each scan were subjected to collision-induced fragmentation if their absolute intensity exceeded 1800 counts. The variable collision energy ranged from 15 to 35 eV depending on the ion’s m/z. Internal calibration used an APCI-TOF (pos) tuning mix introduced to the ion source via a 20-μL loop at the beginning/end of each analysis using a six-port valve. Data were collected and processed by DataAnalysis 4.3 (Bruker Daltonik GmbH, Bremen, Germany). Stock solutions of quercetrin (quercetin 3-rhamnoside), rosmarinic acid and cryptotanshinone were prepared in MeOH at concentrations of 4.2, 5.6 and 4.1 mg/mL, respectively, and kept frozen until used. Calibration curves for these three compounds were constructed based on six concentration points (from 500 ng/mL to 6 μg/mL). The concentrations of the phenolic derivatives in the Herbmix sample were calculated as equivalents of quercetrin (quercetin 3-rhamnoside) or rosmarinic acid, and cryptotanshinone was used to calculate the amount of diterpenoids identified in the extract. All analyses were performed in triplicate.

### Parasitological techniques

Fecal samples were collected on D20, D28, D35, D42, D49, D56, D62 and D70 post-infection and stored at 5 °C in a cooling box until laboratory examination. A modified McMaster technique [21] with a sensitivity of 50 EPG of faeces was used for the detection of strongylid eggs. All animals were humanely killed on D70 and postmortem helminthological dissections were performed. The abomasum was removed and opened, and the contents were emptied into a bucket. The abomasal mucosa was washed gently with water, washing the parasites into the bucket. The contents of the bucket were adjusted to two liters and thoroughly mixed. Two aliquots of 100 mL were then taken, and the numbers of *H. contortus* in each aliquot were counted.

### Hematological parameters

Samples of blood were collected from the jugular vein of each animal on D7, D35, D49 and D70 using a 21-gauge needle and syringe and were placed into microtubes containing 1.6 mg/mL EDTA-K3 (Sarstedt AG & Co, Nümbrecht, Germany). Hematological parameters (red blood cells, hemoglobin, hematocrit, total leukocytes, lymphocytes, monocytes, eosinophils and basophils) were determined immediately by an automated hematological analyzer (Abbott CELL-DYN 3700, Global Medical Instrumentation, Inc., Ramsey, USA). Blood samples for sera were collected into 10-mL serum-separator tubes (Sarstedt AG & Co, Nümbrecht, Germany) and centrifuged at 1200 *g* for 10 min at room temperature. The sera were stored at − 80 °C until analysis.

### Inflammatory response

The concentrations of serum amyloid A (SAA) and serum calprotectin were determined using commercial sheep ELISA kits (MyBioSource Ltd., San Diego, USA). Undiluted serum samples were analyzed following the manufacturer’s instructions. The SAA and calprotectin concentrations had coefficients of variation < 10 and < 15% (intra- and interassay), respectively. The sensitivities of the Elisa kits were 0.1 ng/mL for calprotectin and 0.1 μg/mL for SAA. The detection range of the SAA kit was 3.12–100 μg/mL. All samples were analyzed in duplicate. The optical density of the samples was determined at 450 nm using an Apollo 11 LB913 Elisa absorbance reader (Berthold Technologies GmbH & Co. KG, Bad Wildbad, Germany). We analyzed both parameters on D7, D35 and D49. Serum immunoglobulin A (IgA) was measured with a sheep IgA enzyme-linked immunosorbent assay (Sheep Immunoglobulin A ELISA Kit, Cusabio, Wuhan Huamei Biotech Co., LTD, Wuhan, China). The sensitivity of the kit was 1.87 μg/mL. A HydroSpeed microplate washer (Tecan Austria GmbH, Grödig/Salzburg, Austria) was used for improving assay precision. The IgA values were determined on D7, D35, D49 and D70 using a microplate reader, with the same optical density described above.

### Antioxidant status

The total antioxidant capacity (TAC) of the serum was measured by an assay for ferric reducing antioxidant power described by Benzie and Strain [22]. A ferrous sulfate solution was used to create a standard curve, and the results were expressed in mmol Fe^2+^ formed per liter of sample. The activity of glutathione peroxidase (GPx) in the blood was assessed spectrophotometrically as described by Paglia and Valentine [23] using a Ransel kit (Randox Laboratories, Ltd., London, UK). The enzymatic activity was evaluated at 37 °C at a wavelength of 340 nm, and the results are expressed in units per mL of blood. The extent of lipid peroxidation indicated by malondialdehyde (MDA) level using 1, 1, 3, 3-teramethoxypropane (Sigma-Aldrich) as an MDA precursor in the calibration curve was determined following the method of Jo and Ahn [24]. Serum total thiol or sulfhydryl (SH) concentration was determined spectrophotometrically using Ellman’s method, based on the reaction of 5,5′-dithio-bis (2-nitrobenzoic acid) with protein thiol groups measured at 412 nm [25]. The concentration of SH groups was calculated using reduced glutathione as the standard, and the results are expressed in mmol/L.

### Mineral status

The mineral content in the diet substrates and the serum concentrations of zinc, iron and copper in the lambs were determined by flame atomic absorption spectrometry in an air-acetylene flame, with deuterium background correction [26], using an AA-7000 atomic absorption spectrophotometer (Shimadzu Co., Kyoto, Japan). Certified lyophilized human plasma, ClinCheck Control (Recipe, Munich, Germany), was used to determine the precision of the analysis.

### Calculations and statistical analysis

Calprotectin, SAA and IgA concentrations were calculated by a four-parameter logistic curve fit (GraphPad Prism, GraphPad Software, Inc., San Diego, USA). Analyses of variance (ANOVAs) (GraphPad Prism, GraphPad Software, Inc., San Diego, USA) were used for analyzing initial body weights (BWs), live-weight gains (LWGs), hematological parameters, inflammatory response, antioxidant status and mineral status as repeated-measures mixed models representing the four animal groups (Control, Herbmix, Zn and Herbmix+Zn) and sampling days. Effects included in the model were treatment, time and the interaction between treatment and time. Student’s *t*-tests were applied to assess the differences between mean egg outputs (EPGs) on different sampling days and worm counts at dissection (Fig. 2, Fig. 3). Results were considered statistically significant at *P* < 0.05.

## Results

### Bioactive compounds

**Fig. 1:**
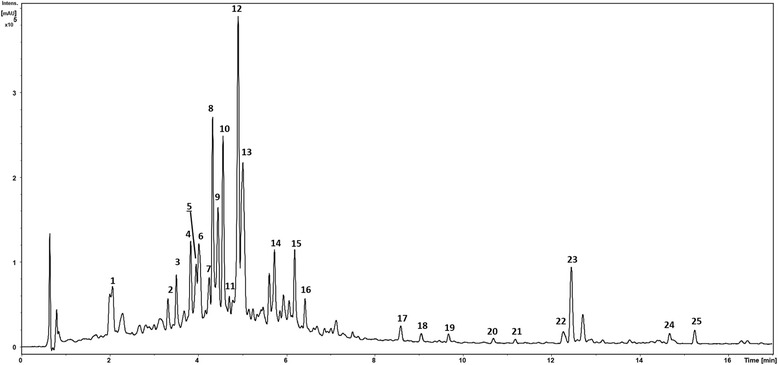
UV chromatogram at 190–600 nm of a sample of Herbmix at a concentration of 100 mg/ml. Peak numbers represent main compounds listed (No.) in Table 2

Quantitative analyses of the bioactive compounds in Herbmix (Table 2) identified three main groups: flavonoids (9964.7 μg/g), diterpenes (4886.1 μg/g) and phenolic acids (3549.2 μg/g). Phenolic acids are Nos. 1 and 12; flavonoids are Nos. 2, 3, 4, 5, 6, 7, 8, 9, 10, 11, 13, 14, 15, 16 and 19 and diterpenes are Nos. 17, 18, 20, 21, 22, 23, 24 and 25. Peak numbers in Fig. 1 represent the main compounds as numbered in Table 2.

**Table 2:** Contents of the main bioactive compounds identified in Herbmix analyzed in positive and negative ionization modes

No.	Compound	RT (min)	UV	*m/z* [M-H]^−^	Formula	MS fragment	*m/z* [M + H]^+^	MS fragment	μg/g DM
1	Chlorogenic acid	2.05	215/325	353.0880	C_16_H_18_O_9_	191	355.1015	163	663.1
2	Quercetin-O-Hex-Hex	3.33	200/335	625.1419	C_27_H_30_O_17_	301	627.1545	303/465/161	134.5
3	Hypolaetin-O-Hex	3.50	255/345	463.0890	C_21_H_20_O_12_	301	465.1016	303	298.5
4	Luteolin 7-glucuronide	3.84	220/255/345	461.0730	C_21_H_18_O_12_	285/163	463.0865	287/257	422.2
5	Quercetin-O-Hex	3.95	265/340	463.0890	C_21_H_20_O_12_	301	465.1018	303/382/141	573.1
6	Quercetin-O-Hex-Pent	4.05	220/350	595.1309	C_26_H_28_O_16_	301	597.1437	303/465	430.9
7	Quercetin-O-Hex-dHex	4.2	220/255/345	609.1466	C_27_H_30_O_16_	301	611.1599	303/465	1436.7
8	Verbascoside	4.340	195/330	623.1985	C_29_H_36_O_15_	461/315/161	625.1756	471/325/163	1162.9
9	3,5-Dicaffeoyl-quinic acid	4.450	200/325	515.1198	C_25_H_24_O_12_	191/353	517.1339	163/319	1602.6
10	Quercetin-O-dHex-dHex	4.553	260/345	593.1512	C_27_H_30_O_15_	–	595.1645	303/449	1441.1
11	Luteolin-glucuronide	4.951	210/340	461.0726	C_21_H_18_O_12_	285	463.0868	287/299	1058.0
12	Rosmarinic acid	4.955	200/330	359.0770	C_18_H_16_O_8_	161/197/179	361.0912	163/181	2886.1
13	Luteolin 3′-(3″-acetylglucuronide)	5.620	270/335	503.0831	C_23_H_20_O_13_	285/255	505.0976	287	296.9
14	Luteolin 3′-(4″-acetylglucuronide)	5.740	270/335	503.0834	C_23_H_20_O_13_	285	505.0977	287	521.7
15	Luteolin-(malonyl-Pent)	6.190	270/335	503.0833	C_23_H_20_O_13_	285	505.0978	287	352.7
16	Isomargaritene	6.425	270/330	591.1709	C_28_H_32_O_14_	283/268/163	593.1862	285/447	221.6
17	Rosmanol	8.5	275/330	345.1704	C_20_H_26_O_5_	301/283	347.1845	301/283/273/231	612.7
18	Isorasmanol or Royleanonic acid	9.05	220/335	345.1705	C_20_H_26_O_5_	283/268/227	347.1846	301/273/259	34.1
19	Acacetin	9.68	220/340	283.0611	C_16_H_12_O_5_	268	285.0752	–	11.3
20	Royleanonic acid-derivative	10.7	–	345.1710	C_20_H_26_O_5_	283/268/227	347.1848	301/273/259	296.8
21	Carnosol derivative	11.25	–	329.1753	C_20_H_26_O_4_	285	331.1901	285/267/303/243	96.1
22	Komaroviquinone	12.3	220	359.1859	C_21_H_28_O_5_	–	361.2012	299/233	1127.1
23	Carnosol	12.5	205	329.1754	C_20_H_26_O_4_	285	331.1899	285/267/303/243	1356.6
24	Carnosic acid	14.6	225	331.1908	C_20_H_28_O_4_	287/245	333.2048	287/245	651.5
25	12-Hydroxy-11-methoxy-8,11,13-abietatrien-20-oic acid	15.3	230	345.2066	C_21_H_30_O_4_	301/286	347.2206	301/219	711.2

### Parasitological status

**Fig. 2:**
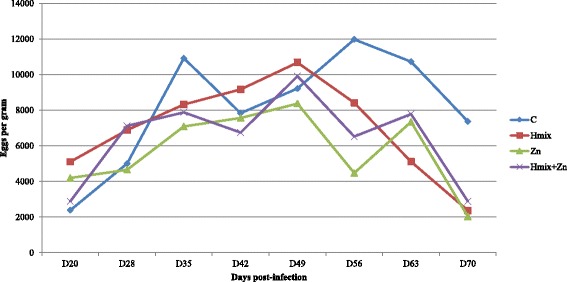
Mean fecal egg counts of the groups of lambs infected with *Haemonchus contortus*

**Fig. 3:**
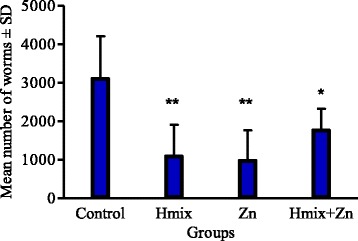
Abomasal worm counts of *Haemonchus contortus* in the lambs of each treatment at the end of the experiment

All lambs had similar BWs (Table 3). Time (*P* < 0.001) affected BW and the mean cumulative LWG. The patterns of shedding of eggs in C, Hmix, Zn and Hmix+Zn are shown in Fig. 2. Data from D49 were used to determine the reduction in egg output in Hmix, Zn and Hmix+Zn relative to C. The mean fecal egg count for all groups increased until D49, with no significant differences between groups (*P* > 0.05), but the egg counts in the lambs treated with Herbmix, zinc and a combination of both decreased after D49. Zn and Hmix+Zn EPGs were significantly lower by D56 (*P* < 0.05 and *P* < 0.01, respectively). EPGs on D70 were significantly lower in all treated groups compared to C (*P* < 0.01). The results of the necropsy on D70 are shown in Fig. 3. Mean worm counts 70 d post-infection were significantly lower in Hmix, Zn and Hmix+Zn than in C (*P* < 0.05 and *P* < 0.01).

**Table 3:** Body weight and mean live-weight gain of lambs infected with *H. contortus* in the experimental groups

Parameter	Day	C	Hmix	Zn	Hmix+Zn	SD	Significance of effects		
							Treatment	Time	Treatment × time
BW	7	14.8	14.8	14.8	15.4	1.72	NS	*	NS
(kg)	35	17.9	18.1	18.6	19.2	1.87			
	49	19.4	20.0	20.9	20.7	1.95			
	70	22.3	22.8	24.1	23.4	2.0			
LWG	35	3.15	3.28	3.82	3.78	1.01	NS	*	NS
(kg)	49	1.50	1.93	2.32	1.55	0.53			
	70	2.92	2.72	3.20	2.68	0.67			

*C* control, *Hmix* Herbmix, *Zn* Zn-glycinate, *Hmix + Zn* Herbmix and Zn-glycinate, *BW* body weight, *LWG* live-weight gain, *NS* not significant

*P < 0.001

### Hematological parameters

The complete red blood cell (RBC) hemograms of each infected animal identified clinical signs of haemonchosis such as anemia from D35 (Table 4). RBC count, hemoglobin (HGB) level and hematocrit (HCT) were influenced by treatment (*P <* 0.001) and time (*P <* 0.001). The treatment and time affected white blood cell (WBC) levels (*P <* 0.05), but differential counts were not affected (*P* > 0.05).

**Table 4:** Hematological parameters of lambs infected with *H. contortus* in the experimental groups

Parameter	Day	C	Hmix	Zn	Hmix+Zn	SD	Significance of effects		
							Treatment	Time	Treatment × time
RBC	7	9.89	11.0	10.8	10.3	0.992	***	***	NS
(T/L)	35	5.94	6.34	6.98	6.31	0.766			
	49	6.23	7.14	8.07	7.21	0.725			
	70	7.52	8.56	9.18	8.65	1.101			
HGB	7	97.1	103.0	97.4	97.8	11.13	***	***	NS
(g/L)	35	58.0	64.6	72.3	61.8	10.40			
	49	58.2	76.8	80.2	69.2	8.09			
	70	72.0	87.1	90.0	81.8	9.981			
HCT	7	0.213	0.220	0.225	0.224	0.017	***	***	NS
(L/L)	35	0.141	0.161	0.182	0.157	0.022			
	49	0.138	0.186	0.195	0.172	0.019			
	70	0.168	0.206	0.207	0.199	0.019			
WBC	7	8.3	10.2	7.2	8.1	3.36	*	NS	NS
(g/L)	35	6.1	9.9	6.9	8.7	2.40			
	49	7.2	8.4	5.9	6.6	1.89			
	70	7.1	7.5	6.7	7.4	1.97			
LYM	7	2.0	2.6	2.1	2.8	1.24	NS	NS	NS
(g/L)	35	2.0	3.5	1.4	2.3	1.23			
	49	2.3	3.1	2.3	1.8	1.13			
	70	2.3	2.7	3.1	2.7	1.23			
MON	7	2.4	2.2	1.6	2.5	0.76	NS	NS	NS
(g/L)	35	1.8	1.9	2.5	1.9	0.74			
	49	1.9	1.7	1.8	2.1	0.70			
	70	2.0	1.9	1.6	1.8	0.62			
EOS	7	0.224	0.092	0.036	0.183	0.2185	NS	NS	NS
(g/L)	35	0.101	0.034	0.141	0.048	0.1075			
	49	0.085	0.069	0.072	0.076	0.0378			
	70	0.297	0.117	0.178	0.165	0.1937			
BAS	7	0.400	0.576	0.377	0.293	0.3002	NS	NS	NS
(g/L)	35	0.347	0.334	0.482	0.328	0.1963			
	49	0.290	0.380	0.270	0.343	0.2675			
	70	0.399	0.395	0.290	0.267	0.2675			

*C* control, *Hmix* Herbmix, *Zn* Zn-glycinate, *Hmix + Zn* Herbmix and Zn-glycinate, *RBC* red blood cells, *HGB* hemoglobin, *HCT* hematocrit,

*WBC* total leukocytes, *LYM* lymphocytes, *MON* monocytes, *EOS* eosinophils, *BAS* basophils, *NS* not significant

* *P* < 0.05, ** *P* < 0.01, *** *P* < 0.001

### Inflammatory response

Mean SAA concentration ranged from 13.7 to 23.7 μg/mL (Table 5). Effect of treatment and the interaction of treatment and time were observed among the groups and sampling days (*P* < 0.05). Mean serum calprotectin concentration ranged from 41.9 to 50.0 ng/mL. Calprotectin concentrations were not influenced by treatment, time and treatment × time (*P* > 0.05). Serum IgA levels were influenced by treatment (*P <* 0.01).

**Table 5:** Inflammatory response in lambs infected with *H. contortus* in the experimental groups

Parameter	Day	C	Hmix	Zn	Hmix+Zn	SD	Significance of effects		
							Treatment	Time	Treatment × time
SAA	7	13.7	18.8	17.6	20.7	5.10	*	NS	*
(μg/mL)	35	17.9	15.4	23.7	14.2	5.20			
	49	17.9	16.9	22.9	18.6	4.12			
Calpro	7	49.1	49.1	48.5	44.7	4.70	NS	NS	NS
(ng/mL)	35	46.9	38.5	50.0	49.0	9.93			
	49	41.9	42.4	48.3	45.9	10.86			
IgA	7	0.287	0.234	0.328	0.211	0.0642	**	NS	NS
(mg/mL)	35	0.309	0.298	0.369	0.309	0.0572			
	49	0.224	0.315	0.360	0.204	0.0842			
	70	0.289	0.318	0.415	0.313	0.1124			

*C* control, *Hmix* Herbmix, *Zn* Zn-glycinate, *Hmix + Zn* Herbmix and Zn-glycinate, *NS* not significant, *SAA* serum amyloid A, *Calpro* calprotectin, *IgA* immunoglobulin A

* *P* < 0.05

### Antioxidant status

The treatment influenced MDA (*P* < 0.05) and SH groups (*P <* 0.01) of lambs (Table 6).

**Table 6:** Antioxidant status in lambs infected with *H. contortus* in the experimental groups

Parameter	Day	C	Hmix	Zn	Hmix+Zn	SD			
							Treatment	Time	Treatment × time
GPx	7	41.2	50.8	45.4	44.2	13.21	NS	***	NS
(U/mL)	35	20.3	23.4	29.3	24.7	8.59			
	49	25.9	25.4	34.7	21.6	6.07			
	70	28.1	40.1	42.8	30.4	8.20			
MDA	7	0.234	0.214	0.209	0.196	0.063	*	**	NS
(μmol/L)	35	0.225	0.225	0.214	0.188	0.046			
	49	0.259	0.248	0.271	0.267	0.049			
	70	0.299	0.195	0.190	0.217	0.060			
TAC	7	0.292	0.254	0.276	0.258	0.042	NS	***	NS
(mmol/L)	35	0.292	0.290	0.262	0.288	0.029			
	49	0.319	0.318	0.309	0.338	0.025			
	70	0.337	0.340	0.333	0.347	0.029			
SH groups	7	0.291	0.287	0.299	0.296	0.030	**	***	NS
(mmol/L)	35	0.329	0.369	0.352	0.344	0.034			
	49	0.316	0.333	0.390	0.332	0.037			
	70	0.326	0.347	0.388	0.368	0.050			

*C* control, *Hmix* Herbmix, *Zn* Zn-glycinate, *Hmix + Zn* Herbmix and Zn-glycinate, *GPx* blood glutathione peroxidase, *MDA* serum malondialdehyde, *TAC* total antioxidant capacity, *SH* sulfhydryl, *NS* not significant

* *P* < 0.05, ** *P* < 0.01, *** *P* < 0.001

All antioxidant indices, i.e. GPx activity (*P* < 0.001), MDA concentration (*P* < 0.01), TAC (*P* < 0.001) and serum SH levels (*P* < 0.001), were influenced by time.

### Mineral status

The serum concentrations of zinc (*P* < 0.001), iron (*P* < 0.01) and copper (*P* < 0.001) were influenced by time (Table 7). An interaction of treatment × time was identified for the zinc concentration (*P* < 0.001).

**Table 7:** Mineral status of serum zinc, iron and copper concentrations (mg/L) in lambs infected with *H. contortus* in the experimental groups

Element	Day	C	Hmix	Zn	Hmix+Zn	SD	Significance of effects		
							Treatment	Time	Treatment × time
Zinc	7	0.456	0.487	0.539	0.527	0.038	NS	**	**
	35	0.727	0.651	1.004	0.759	0.153			
	49	0.751	0.693	0.834	0.740	0.059			
	70	0.773	0.833	0.823	0.871	0.041			
Iron	7	1.370	1.409	1.301	1.306	0.052	NS	*	NS
	35	0.970	1.267	1.537	1.151	0.238			
	49	0.876	1.351	1.351	1.270	0.228			
	70	1.284	1.827	1.773	1.744	0.251			
Copper	7	0.601	0.641	0.637	0.701	0.041	NS	**	NS
	35	0.764	0.787	0.943	0.861	0.081			
	49	0.753	0.759	0.814	0.816	0.034			
	70	0.764	0.861	0.844	0.826	0.042			

*C* control, *Hmix* Herbmix, *Zn* Zn-glycinate, *Hmix + Zn* Herbmix and Zn-glycinate, *NS* not significant * *P* < 0.01, ** *P* < 0.001

## Discussion

### Bioactive compounds

Tannin-rich herbs have direct antiparasitic activity against internal nematodes in ruminants and can indirectly increase host resistance [9, 10]. Traditional medicines made from medicinal plants represent a source of multitarget therapeutics, and their bioactive compounds work synergistically [7, 27]. The UHRMS analysis of the bioactive compounds in Herbmix identified mainly flavonoids (54%) diterpenes (27%) and phenolic acids (19%) (Table 2). Flavonoids can mitigate diseases associated with oxidative stress by their antioxidant properties [7, 28] and also probably act via a mechanism similar to that of tannins [29]. Quercetin [28, 30], verbascoside [31, 32] and luteolin [33] were the most abundant Herbmix flavonoids with antioxidant and anti-inflammatory properties. Luteolin and quercetin can inhibit the motility of *Trichostrongylus colubriformis* larvae [34] and larval exsheathment in *H. contortus* [35]. These flavonoids, however, also synergistically increase the activity of condensed tannins [35]. Carnosic acid and carnosol, which are typical for rosemary, were highly abundant diterpenes in Herbmix, and both have antioxidant activity [36]. Rosmarinic acid, however, was the most bioactive substance in Herbmix. The two phenolic acids in Herbmix, rosmarinic acid [37, 38] and chlorogenic acid [39–41], both have anti-inflammatory and antioxidant biological activities with beneficial health-promoting effects.

### Parasitological status

The effect of Herbmix on BW and LWG in the infected lambs previously observed by Váradyová et al. [42] was not confirmed in the present experiment. Many studies have confirmed the negative impact of gastrointestinal nematode infections on sheep performance. The results of a meta-analysis indicated that weight gain in animals infected with *H. contortus* was 77% of the gain in parasite-free animals [43]. Most of the trials in the meta-analysis reported a negative effect of parasitism on production, but the effect was significant in only 58.3% of the trials. EPGs in the treated groups in our study decreased significantly only toward the end of the experiment, after D56. Obtaining significant LWGs in the treated groups in last 14 days of experiment was thus not surprising.

Egg output began to decrease in all three treated groups after D49, with reductions > 70% between D49 and D70. Two conclusions can be drawn. Firstly, no treatment effect has been documented, because egg output did not change until D49. This is in contrast with our previous results obtained by Váradyová et al. [42] where egg output decreased by D35. However, Herbmix they used contained more medicinal herbs including also *Artemisia absinthium* and the anthelmintic activity of *Artemisia* species against the ovine nematodes has well been documented [44–46]. Secondly, animals increased their resistance to the worms and expelled adult parasites during the course of infection, supported by the significant reductions of parasites in the treated groups at necropsy. Significant effect of treatment on serum IgA levels pointed out on second conclusion that local antibody response may play important role to immunity of lambs to *H. contortus* infection.

### Hematological parameters

The experimental infection of the four groups of lambs with *H. contortus* caused clinical signs of haemonchosis such as anemia as was described by Bordoloi et al. [47]. RBC and HGB were lower for all four infected groups than the normal ranges of 9.00–15.0 T/L and 90.0–150.0 g/L, respectively [48]. HGB was higher in Hmix and Zn than C. Increased levels of HGB in goats fed a tannin-rich plant mixture containing condensed tannins (1.96%) were described by Jan et al. [49].

### Inflammatory response

The concentrations of SAA, serum calprotectin and IgA were evaluated as markers of inflammation to monitor *H. contortus* infection. SAA is a non-specific inflammatory protein indicating inflammatory disease, injury or infection but is a highly sensitive, effective marker of inflammation in ruminants [50–52]. The effects of gastrointestinal nematodes on SAA concentration have not been extensively studied, but Ulutaş et al. [53] reported an increase in SAA concentrations with mixed gastrointestinal infections of nematodes and liver trematodes. The concentration of SAA in our study was influenced, however no effects were observed in our previous study of lambs infected with *H. contortus* [42]. The response of SAA to the experimental conditions was weak and non-uniform, so we cannot currently consider SAA a useful marker for monitoring *H. contortus* infections in lambs. Calprotectin is a major cytosolic protein of leucocytes, especially neutrophils. Elevated levels of calprotectin are evident in infectious and inflammatory diseases and are often used as a marker of gastrointestinal inflammation [54]. The response of serum calprotectin during *H. contortus* infection and the experimental treatments in our present study was inconsistent with study of Váradyová et al. [42]. Serum IgA in sheep is predominantly derived from the intestine and closely associated with intestinal mucosal immune responses [14]. Cardia et al. [55] reported higher serum IgA levels against L3 in lambs infected with *Trichostrongylus colubriformis*. Increased levels of IgA in naturally infected sheep have been positively associated with resistance to *Teladorsagia circumcincta* by suppressing parasite growth; development and fecundity, mediated by IgA activity against L4 larvae [56, 57]. Serum IgA levels in our study were influenced by treatment, however the immune response against *H. contortus* was very similar in all experimental groups.

### Antioxidant status

Several studies have reported that medicinal plants have a wide range of antioxidant capacities and that phenolic compounds are a major contributor to the antioxidant activity of these plants, making them promising sources of natural antioxidants [58]. Herbmix containing predominantly flavonoids (54%) exhibited antioxidant potential in vivo [7] by reducing the MDA level, indicating a decrease in lipid peroxidation in the serum, and tending to increase GPx activity in the blood of infected lambs. Zinc can exert its antioxidant action by several possible mechanisms, e.g. the protection of protein SH groups from oxidation. Zinc is also involved in the synthesis of molecules rich in SH groups, such as reduced glutathione and metallothionein, which play an antioxidative role [59]. The levels of SH groups on D49 and D70 indicated that zinc supplementation improved the protection of various thiols in the infected lambs by increasing serum SH levels. The activity of blood GPx was higher, and lipid peroxidation in the serum was lower, at the end of the experiment in the groups treated with zinc than in the untreated group. Pivoto et al. [60] also demonstrated that zinc could help reduce the oxidative stress caused by *H. contortus* in lambs. Our findings indicate that treatment with organic zinc alone or in combination with herbal nutraceuticals could potentially provide natural antioxidants for minimizing oxidative stress in nematode infected lambs.

### Mineral status

Experimental infection with *H. contortus* can decrease mineral concentrations in the liver or serum of lambs [42, 61]. Concentration of serum zinc of lambs in our treatments was not influenced. The efficiency of immunological responses against gastrointestinal nematodes depends on the zinc intake of the host, supporting the important role of zinc in the maintenance of the gut epithelial barrier and intestinal immunity [62]. Our unsupplemented control lambs had marginal serum levels of iron, associated with their low blood HGB levels, but supplementation with zinc or Herbmix maintained higher serum zinc and iron levels, providing better conditions for the lambs to cope with this parasitic disease and reduce parasitic burdens.

## Conclusion

This study did not fully confirm a direct anthelmintic impact on the viability of nematodes, but the treatments with herbal nutraceuticals, zinc and both diet supplements together likely indirectly contributed to an increase in the resistance of lambs to nematode infections. The bioactive compounds identified in Herbmix have anti-inflammatory and antioxidant biological activities with beneficial health-promoting effects. The Herbmix, organic zinc and combination of Herbmix with organic zinc contributed to reduction of nematode parasitic infection in experimental animals. The use of herbs with bioactive compounds together with some essential trace elements as alternatives to conventional anthelmintics can be useful for economical, effective and sustainable animal production.

## Abbreviations

ANOVA: analysis of variance
BW: body weight
C: control
Calpro: calprotectin
D: day of sample collection
DM: dry matter
EPG: eggs per gram
GPx: glutathione peroxidase
HCT: hematocrit
HGB: hemoglobin
Hmix: Herbmix
IgA: immunoglobulin A
LWG: live-weight gain
MDA: malondialdehyde
PSM: plant secondary metabolites
RBC: red blood cells
SAA: serum amyloid A
SH: sulfhydryl
SPE: solid-phase extraction
TAC: total antioxidant capacity
UHRMS: ultra-high resolution mass spectrometry
